# Analysis of the most influential publications on vertebral augmentation for treating osteoporotic vertebral compression fracture: A review

**DOI:** 10.1097/MD.0000000000030023

**Published:** 2022-08-05

**Authors:** Shuang Wang, Liang Zheng, Jun-Xiong Ma, Hong Wang, Shao-Tong Sun, Bo-Hua Zhang, Xin-Lei Guo, Liang-Bi Xiang, Yu Chen

**Affiliations:** a Department of Orthopedics, General Hospital of Northern Theater Command, Shenyang, China.

**Keywords:** bibliometric study, kyphoplasty, percutaneous vertebral augmentation, vertebroplasty

## Abstract

This study aimed to analyze the most influential publications on vertebral augmentation for treating osteoporotic vertebral compression fracture. The Web of Science database was searched using the key words “percutaneous vertebroplasty,” “percutaneous kyphoplasty,” “balloon kyphoplasty,” “vertebroplasty,” “kyphoplasty,” and “vertebral augmentation.” The top 100 publications were arranged by citations per year and descriptively and visually analyzed. The top 100 publications were cited 25,482 times, with an average of 14.4 citations per paper per year. The corresponding authors of the publications represented 17 nations, with most authors being American (46 authors). Thirty-two journals were involved, with *SPINE* issuing the most publications (24 papers of the 100). Clinical research (73 of the 100 papers) outnumbered basic studies (14 papers) and systematic reviews (13 papers), and the most publications were published between 2000 and 2004. Co-citation analysis of the key words indicated that the top 5 focus areas were “complication,” “balloon kyphoplasty,” “vertebral compression fracture,” “biomechanics,” and “calcium phosphate cement.” The top 3 keywords with the strongest citation bursts were “compression fracture,” “cement,” and “balloon kyphoplasty.” The keywords with persistent strong citation bursts are “balloon kyphoplasty” and “augmentation.” There are still contrary opinions about vertebral augmentation; new research should be conducted with more deliberate design and longer follow-up.

## 1. Introduction

Bibliometric studies refer to the statistical analysis of the particular group of publications that are associated with a specific field or subject. By this method, researchers can comprehend the subject more thoroughly and grasp the evolution of the subject more accurately. Meanwhile, influential authors and institutions can be identified by the number of times they have been cited. Although it has several limitations, the number of citations is still seen as the index that most commonly represents influence. Several bibliometric studies have been published focusing on issues from different perspectives, such as the particular subject,^[1–4]^ different anatomic regions,^[5,6]^ kinds of surgical technologies,^[7]^ special kinds of diseases,^[8,9]^ and even a given territory.^[10]^ In recent years, new methods and software have emerged for the analysis of publications. More sensitive algorithms have been applied to reveal the relationships between publications, authors, institutions, and even countries. By these means, past trends, present hotspots, and future prospects could all be explored and exhibited. These new methods also enabled the ability to find the burst points of traditional research. The results of these analyses can be visualized using software such as CiteSpace and VOSviewer.

Vertebral augmentation is a type of operation used to restore the strength and stiffness of the vertebra after fractures caused by tumors, trauma, or osteoporosis. Since this technique was introduced by Galibert et al^[11]^ in 1987, it has been used extensively to treat several traumas and diseases, particularly osteoporotic vertebral compression fractures (OVCFs). Generally, vertebral augmentation includes 2 kinds of operations: percutaneous vertebroplasty (PVP) and percutaneous kyphoplasty (PKP). The core purpose of both operations is to insert bone cement into the vertebral body impacted by the fracture. Many clinical trials and basic research studies have attempted to explore the technology, indication, results, prognosis, and other factors associated with vertebral augmentation. Although most clinical trials exhibited excellent results in terms of pain relief and function restoration, some results were contradictory. Meanwhile, complications related to vertebral augmentation were observed, especially fatal pulmonary embolisms. In 2009, 2 level I evidence publications indicated that vertebral augmentation, especially PVP, was not more efficacious than conservative treatment when treating OVCF.^[12,13]^ Both articles have been frequently cited thereafter. Based on these 2 articles and other level II evidence publications,^[14–16]^ the American Academy of Orthopaedic Surgeons (AAOS)^[17]^ strongly recommended against PVP for treating OVCFs diagnosed on imaging with correlating clinical signs, whereas PKP was weakly recommended for patients with similar diagnoses.

Even with the above AAOS recommendation, vertebral augmentation was still used in clinical practice. Many patients, especially some elderly patients, benefited markedly from these operations. Many new materials were invented to replace the traditional bone cement in order to overcome the disadvantages, and several new methods of operation were discussed, such as special puncturing routes. All these materials and technologies made the operations safer and more accurate. However, since there were still conflicting opinions about these technologies, it seemed necessary for researchers to explore vertebral augmentation further, especially the indications of this kind of operation.

The treatment could present optimal results only when it was used to treat the most suitable patients. The details of the method, including the technology used in the course of the operation, the rehabilitation method used after the operation, and the characteristics of materials used to fill the vertebral body, should also be discussed. For these discussions, the publications that have been published in the last few years should be reviewed, with the most frequently cited articles analyzed using bibliometrics. By these means, the results could be assessed from different perspectives and exhibited visually using professional charts and figures.

In this review, the most influential publications about vertebral augmentation, including PVP and PKP, were searched mainly in the database of the Web of Science Core Collection and analyzed. Additionally, the information in the publications was collected and researched thoroughly. The authors attempted the following: identify the 100 papers about vertebral augmentation that were cited most frequently; review the content of the papers to determine the focus areas in the field; analyze the internal relationships between the articles, such as the key word co-occurrence, paper co-citation, and key words time-zone. After discussing the results of the above analyses, the authors propose some suggestions about vertebral augmentation for consideration by researchers.

## 2. Materials and Methods

In August 2021, the databases of the Web of Science Core Collection were searched using the keywords “percutaneous vertebroplasty,” “percutaneous kyphoplasty,” “balloon kyphoplasty,” “vertebroplasty,” “kyphoplasty,” and “vertebral augmentation.” Then, all the publications were reviewed by the first author and the co-first authors of the current study. The inclusion criteria of the publications were as follows: be associated with vertebral augmentation operations such as PVP or PKP, be searchable in the Web of Science Core Collection database because the information of this database was intact and suitable for the visual software analysis, and be published in English. Meanwhile, the exclusion criteria were as follows: be associated with other content such as the treatment principle of OVCF or discussion about the use of bone cement in the field of orthopedics; conference papers, letters, or other informal articles; and published in languages other than English. All articles searched were reviewed by the 3 coauthors separately to exclude the articles that were not closely associated with PVP or PKP. For example, research about the general treatment principles of osteoporotic fracture or some studies about the properties of bone cement were excluded. When there were differences in opinions about some articles, the corresponding authors discussed the articles with all authors and made the final decision. Since this research was a review of the publications and no patients were included, the requirement for ethical approval was waived.

After determining the publications to be included in the analysis, the number of times a publication was cited per year (total number of times cited/years from publication to 2021) was calculated, and the top 100 papers were defined as the most influential publications about vertebral augmentation.

The collected data included the journal and the year of publication, the nation of the corresponding author, total number of times cited, the number of times cited per year since publication, and the study type. When the publications were clinical studies, the evidence of the study was graded between 1 and 5 following the guidelines of the Oxford Centre for Evidence-Based Medicine (March 2009). Then, systematic reviews and meta-analyses were combined as a specific type. A visual analysis of the 100 papers was conducted using the CiteSpace software.

## 3. Results

### 3.1. General information about the search strategy

Using the keywords mentioned above, a total of 6621 papers were found. Then, 2271 papers were selected by the screening step using the inclusion and exclusion criteria. The 100 most influential papers were then determined based on the number of citations per year, and the top 10 publications are listed in Table 1.

**Table 1:** Top 10 publications with the most citation times per year.

Rank	Title	Type	Authors	Journal	Publication year	Total citations	Average per year
1	A randomized trial of vertebroplasty for osteoporotic spinal fractures	Randomized controlled trial	Kallmes et al^[12]^	*New England Journal of Medicine*	2009	870	66.92
2	A randomized trial of vertebroplasty for painful osteoporotic vertebral fractures	Randomized controlled trial	Buchbinder et al^[13]^	*New England Journal of Medicine*	2009	859	66.08
3	Vertebroplasty versus conservative treatment in acute osteoporotic vertebral compression fractures (Vertos II): an open-label randomised trial	Randomized controlled trial	Klazen et al^[20]^	*Lancet*	2010	522	43.5
4	Efficacy and safety of balloon kyphoplasty compared with non-surgical care for vertebral compression fracture (FREE): a randomised controlled trial	Randomized controlled trial	Wardlaw et al^[27]^	*Lancet*	2009	511	39.31
5	New technologies in spine: kyphoplasty and vertebroplasty for the treatment of painful osteoporotic compression fractures	Review		*SPINE*	2001	729	34.71
6	Initial outcome and efficacy of “kyphoplasty” in the treatment of painful osteoporotic vertebral compression fractures	Clinical trial		*SPINE*	2001	659	31.38
7	Vertebroplasty and kyphoplasty: a systematic review of 69 clinical studies	Review	Hulme et al^[32]^	*SPINE*	2006	483	30.19
8	Percutaneous polymethylmethacrylate vertebroplasty in the treatment of osteoporotic vertebral body compression fractures: technical aspects	Clinical trial	Jensen et al^[18]^	*American Journal of Neuroradiology*	1997	751	30.04
9	Percutaneous vertebroplasty with polymethylmethacrylate. Technique, indications, and results	Review		*Radiologic Clinics of North America*	1998	644	26.83
10	Safety and efficacy of vertebroplasty for acute painful osteoporotic fractures (VAPOUR): a multicentre, randomised, double-blind, placebo-controlled trial	Comment	Clark et al^[19]^	*Lancet*	2016	157	26.17

The 100 most influential publications were cited 25,482 times, and each paper was cited 14.4 times per year on average. The papers were cited 91–870 times, and the number of citations per year ranged from 7.25 to 66.92.

### 3.2. Distribution of publications in journals and countries

The 100 articles were published in 32 journals. The highest number of articles came from *SPINE* at 24, followed by the *European Spine Journal* at 11, and the *American Journal of Neuroradiology* and *Radiology* with 8 each (Table 2).

**Table 2:** Distribution of the publications in journals.

Journal	Number of publications
*Spine*	24
*European Spine Journal*	11
*American Journal of Neuroradiology*	8
*Radiology*	8
*Journal of Bone and Mineral Research*	5
*Journal of Vascular and Interventional Radiology*	5
*Journal of Neurosurgery*	4
*Spine Journal*	4
*Lancet*	3
*Bone*	2
*New England Journal of Medicine*	2
*Osteoporosis International*	2

Corresponding authors came from 17 different nations, among which American authors were most common at 46, followed by French authors at 11. There were as many as 5 authors from Australia, Switzerland, and Korea (Table 3).

**Table 3:** Distribution of the authors in countries.

Country	Number of publications
United States	46
France	11
Australia	5
Switzerland	5
Korea	5
China	4
Germany	4
Netherlands	3
United Kingdom	3
Canada	3

### 3.3. Distribution of level of evidence and year of publication

**Figure 1.**
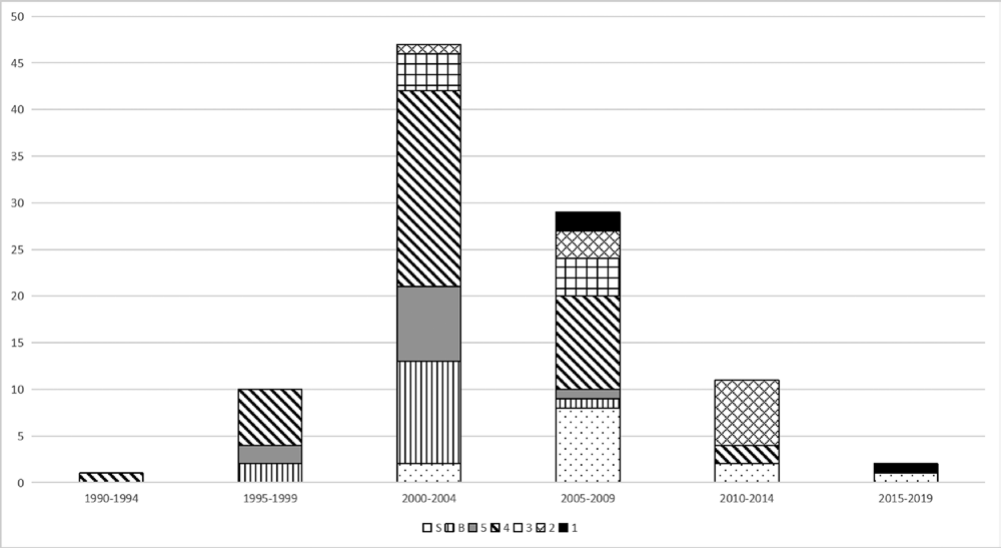
The distribution of different types of publications in years. 1–5 refer to the level of evidence for clinical studies. B means to the basic studies and S for the systemic reviews and meta-analyses.

As for the level of evidence of clinical research, 3 studies met the standard of level I evidence, and 11 publications were level II, while 8 publications were level III. There were 40 level IV and 11 level V studies. The number of basic studies was 14, while that of systematic reviews and meta-analyses was 13. Level 4 evidence studies were most common in the current research area. Most studies were published in the years 2000–2004, with 47 papers published in this period (Fig. 1).

### 3.4. Co-citation analysis of key words

**Figure 2.**
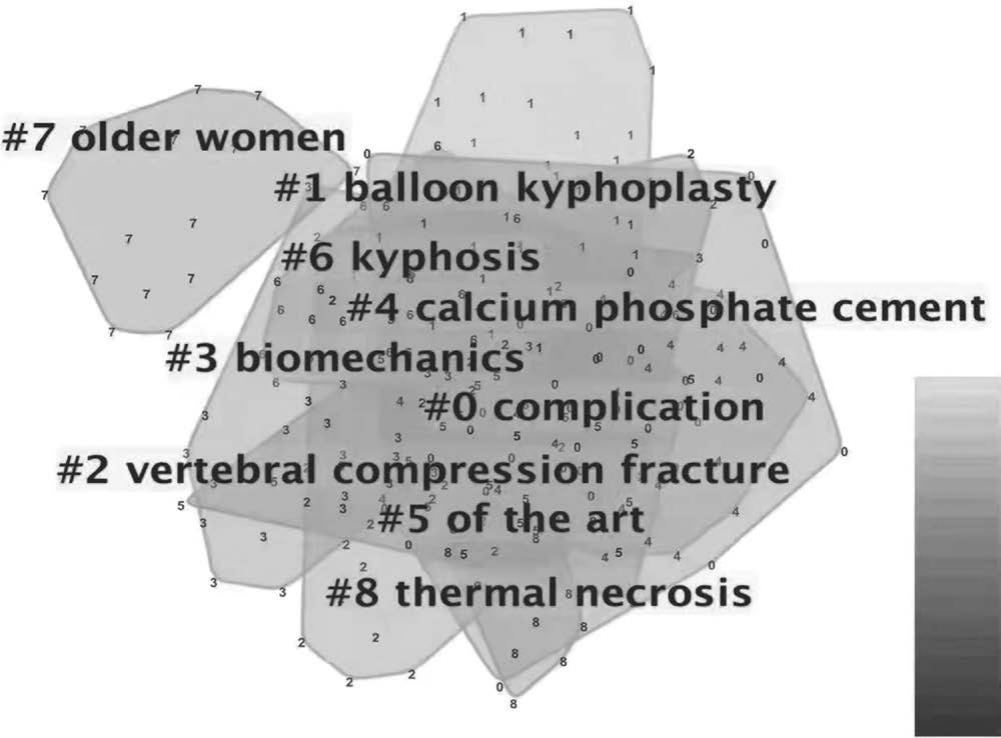
Reference co-citation network for the 100 publications clustered according to key words. PVP = percutaneous vertebroplasty.

After analyzing the key words, co-citation results of the key words could be divided into 15 cluster categories, as shown in Figure 2: #0, complications; #1, balloon kyphoplasty; #2, vertebral compression fracture; #3, biomechanics; #4, calcium phosphate cement; #5, state-of-the-art; #6, kyphosis; #7, elderly women; and #8, thermal necrosis.

**Figure 3.**
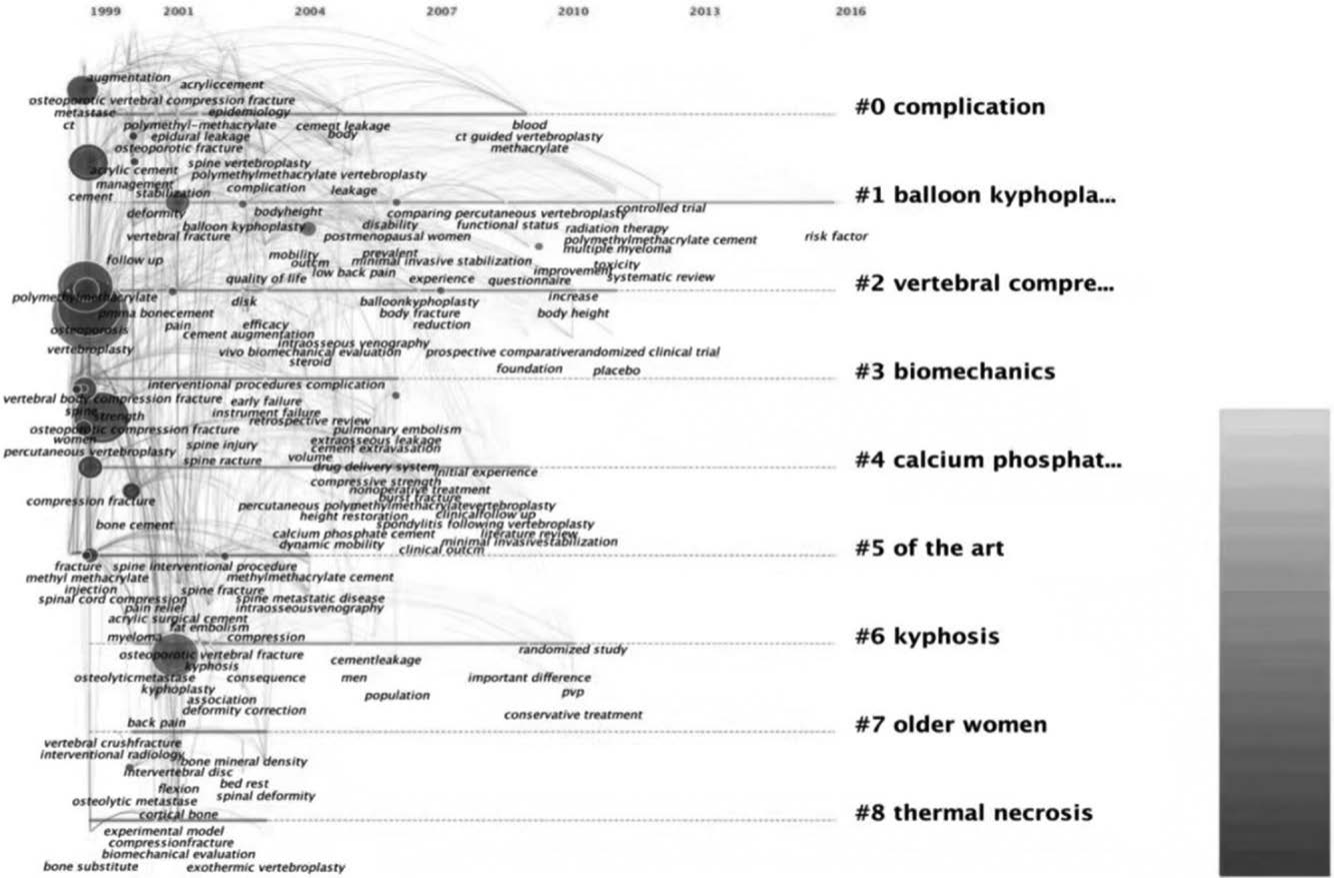
The timeline map of co-cited references according to key words. The 8 identified cluster subfields were listed and the time span were shown with the research progress of the evolution.

The timeline map of co-cited references is shown in Figure 3. The 8 identified cluster subfields were listed, and the time span was shown with the research progress of the evolution.

### 3.5. Keyword citation burst analysis and publication co-citation analysis

**Figure 4.**
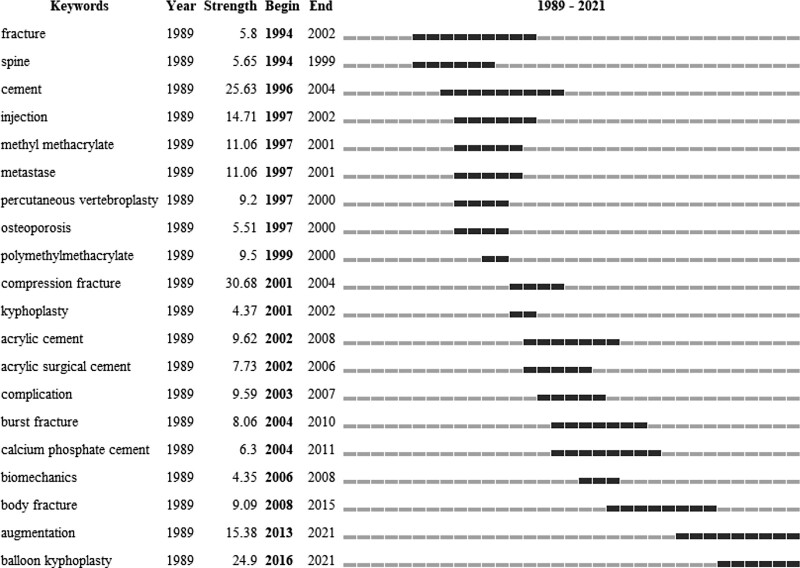
Top 20 keywords with the strongest bursts of citation.

The top 20 keywords with the strongest citation bursts by the beginning of the year are listed in Figure 4. Compression fracture had the highest burst strength of 30.68, cement was second at 25.63, and balloon kyphoplasty was third at 24.9. The citation burst for the keyword balloon kyphoplasty occurred in 2016 and is still continuing. The citation burst for the keyword augmentation has also not ended.

**Figure 5.**
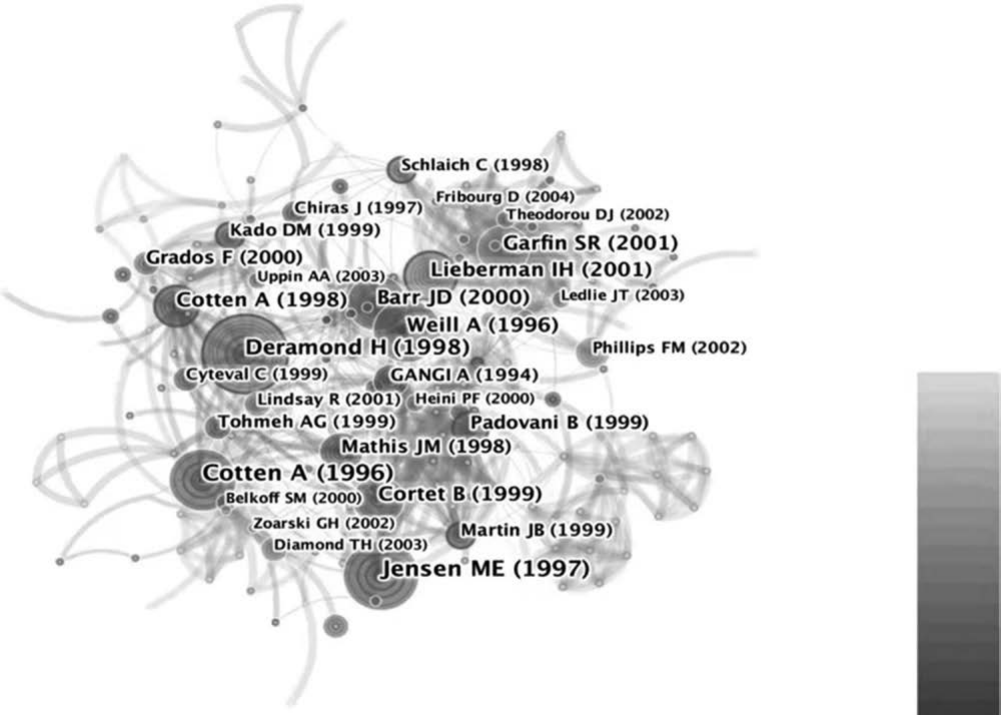
Co-citation analysis of the references.

Co-citation analysis of the references is shown in Figure 5. The publications by Jensen et al^[18]^ was the highly cited reference.

## 4. Discussion

The most frequently cited papers on vertebral augmentation in the treatment of vertebral compression fracture.

In 1987, Galibert et al^[11]^ showed that certain vertebral angiomas could be destroyed through percutaneous intrasomatic injection of acrylic cement, thereby consolidating the vertebral column. Despite only 7 cases being reported and a follow-up period of only 2 years, the authors concluded that this technology was an ideal alternative for treating tumors in vertebrates. This article was seen as the first appearance of PVP, and it became the most frequently cited article, despite being published in French. Ten years later, Jensen et al^[18]^ reported on PVP for treating OVCFs and concluded that the procedure provided immediate pain relief and early mobilization in appropriate patients. This article was the second most frequently cited.

After 1997, the use of PVP and PKP for OVCF treatment spread globally, and much research was conducted to explore its indications, complications, prognosis, and results especially when compared with conservative methods. Some prospective studies and randomized trials were conducted, including that by Kallmes et al^[12]^ in 2009, which was the third most frequently cited, and some confusing results emerged.

### 4.1. Details of the content of papers and opinions of the researchers

In 2009, *The New England Journal of Medicine* published 2 randomized clinical trials (RCTs) about vertebroplasty in the same issue. These 2 RCTs were the 2 most frequently cited publications per year in our research. In the first article, Buchbinder et al^[13]^ performed a multicenter, randomized, double-blind, placebo-controlled trial to compare vertebroplasty and sham procedure in 78 patients >50 years old with painful, unhealed osteoporotic vertebral fractures that occurred <12 months prior to the procedure. The outcomes included overall pain, pain at night and at rest, physical functioning, quality of life, and perceived improvement at 1 week and 1, 3, and 6 months. The authors concluded that vertebroplasty did not have a significant advantage in all measured outcomes at all time points. Additionally, there were no differences in the incidence of vertebral fractures after operations. In this study, patients were randomly assigned to different groups before entering the operating room. For the patients in the sham intervention group, all procedures were similar to those in the PVP group until the needle was inserted into the lamina. Then gentle tapping was also performed to simulate the manipulation of PVP.

In the second study, Kallmes et al^[12]^ compared vertebroplasty with the simulated procedure without cement for osteoporotic spinal fractures in 131 patients, and the follow-up period was 3 months. In their multicenter trial, the modified Roland-Morris Disability Questionnaire and ratings of average pain intensity were used as primary outcomes. The conclusions indicated that patients in the vertebroplasty group and control group experienced similar improvements, and there were no differences between the 2 groups. In this study, the patients were randomized after anesthetizing the skin and subcutaneous tissues. The blinding was implemented by verbal and physical cues; for example, pressure was applied on the back and the odor was similar to that of polymethyl methacrylate.

These 2 articles were important as they changed traditional opinions about PVP and became the top 2 most frequently cited publications. Many surgeons and physicians began proposing conservative treatment for these kinds of operations, especially when they were used to treat osteoporotic spinal fractures. Based on these 2 articles, which were categorized as level I evidence, and the other 3 level II articles,^[14–16]^ the AAOS^[17]^ strongly recommended against vertebroplasty for patients with OVCF based on imaging with correlating clinical signs, and kyphoplasty was recommended weakly for the same patients. The numbers of publications rapidly declined after 2009 resulting in obvious differences in our analysis for nearly all kinds of studies.

Still, there were contrary opinions. Another level I evidence in our research was the study by Clark et al^[19]^ published in 2016. There were 120 patients with acute OVCFs occurring <6 weeks prior to the study. Researchers simulated vertebroplasty by performing all the same procedures until the short needle reached the periosteum; then, skin pressure and tapping on the needle were applied to mimic vertebroplasty. Other factors, such as conversations about polymethyl methacrylate mixing and injection suggestion, were also used to simulate vertebroplasty. The authors concluded that vertebroplasty was superior to conservative treatment for patients with OVCFs that occurred <6 weeks prior to the procedure.

These level I evidence trials used careful designs and great effort to ensure blinding. This was also the most difficult aspect to assess in the evaluation of PVP and PKP versus conservative treatments for OVCF. Most of the publications were graded as level II evidence because of the absence of blinding. In these level II evidence publications, most still compared PVP^[16,20–22]^ or PKP^[23–27]^ with nonsurgical treatments. Nearly all level II evidence studies achieved similar results, indicating that these 2 methods were more suitable for OVCF than conservative treatment. Even a publication with a nearly neutral opinion indicated that PKP can relieve pain more rapidly than conservative treatment.^[14]^ Two other publications compared PVP with PKP; one recommended PVP because of the higher cost of PKP,^[28]^ and the other one concluded that the results of PVP and PKP were similar, with a shorter procedure duration in PVP but fewer cement leakages and longer fracture-free survival in PKP.^[29]^ The last level II evidence publication compared PKP with conservative treatment in patients with vertebral compression fractures (VCF) and cancer and strongly recommended PKP.^[30]^

In our current study, systematic reviews and meta-analyses were combined as a specific kind of publication. There were 13 publications of this kind, all published after 2000, and most (13/15) were published after 2005. It is possible that this distribution indicated shift to stopping or pausing vertebral augmentation.^[31]^

Systematic reviews by different authors recognized the rapid pain relief of PVP and PKP generally, but recommended that comparative, blinded, RCTs should be performed and standardized evaluative methods should be adopted since there was not enough evidence to support the safety and effectiveness of these technologies.^[32–37]^ Some authors also mentioned providing information to the patients about the benefits and potential harms before operations.^[38]^ Taylor et al^[39]^ compared PKP with PVP and concluded that both procedures could achieve benefits in the treatment of OVCF, but PKP appeared to have a better adverse event profile. Other systematic reviews focused on complications such as pulmonary cement embolism.^[40]^ Contrary to these cautious recommendations in the treatment of OVCF, the applications of PVP and PKP for cancer-related VCFs have gained extensive recognition.^[41]^

As for meta-analyses, Eck et al^[42]^ compared PVP and PKP in 2008 and concluded that both methods could provide improvement in visual analog scale pain scores, but PVP had a more significant improvement and also a greater risk of cement leakage and new fractures. Wang et al^[43]^ achieved similar results after comparing PVP and PKP for the treatment of single-level VCF. Lee et al^[44]^ considered PVP and PKP as minimally invasive procedures for VCF and evoked future prospective studies to validate the results. Anderson et al^[45]^ recommended cement augmentation in the treatment of symptomatic VCF. Chen et al^[46]^ also compared PVP and PKP with conservative treatment in elderly patients with OVCF and concluded that PVP achieved the best effect in relieving pain. Conservative treatment was associated with the lowest incidence of new fractures, and balloon kyphoplasty had the lowest risk of all-cause discontinuation.

The 14 basic studies were mostly biomechanical studies using cadaveric vertebral bodies^[47–54]^ or the method of finite-element analysis.^[55–57]^ These studies reached similar conclusions that cement augmentation could restore the strength of the affected vertebrae^[49]^ but could increase the risk for fractures of the adjacent vertebrae. Some technology skills were discussed in these studies. Belkoff et al^[50]^ tested the effect of tamp treatment used in PKP and confirmed that this technology could restore the height of vertebrae better than PVP. Molloy et al^[51]^ related the strength and stiffness with the percentage of cement-filling volume during PVP. Steinmann et al^[54]^ compared unipedicular with bipedicular approaches and recommended the former for comprehensive consideration. Experimental models^[58]^ and cadaveric vertebral bodies^[52,53]^ were also used to study the properties of some special kinds of cement or to compare different kinds of cement.

Most clinical studies were level III-V evidence publications, including case-control studies, retrospective comparative studies, case series reports, and reviews. The numbers of these publications decreased after 2009. These studies discussed PVP and PKP from different perspectives and reported different results about these 2 procedures. Nearly all studies were associated with OVCF treatment, and only 9 were about the use of these operations to treat vertebral fractures with tumors, such as angiomas,^[11]^ multiple myeloma,^[59]^ or metastases.^[60]^ The follow-up period was within 1–24 months, and the number of cases reported was up to a hundred. The longest observation period was in a cohort study of Edidin et al^[61]^ at 4 years. This study indicated that patients in the nonoperated cohort had a lower adjusted survival rate than patients in the operation group. Meanwhile, kyphoplasty had a lower relative risk of mortality than PVP. This showed that longer follow-up might indicate different results for these procedures, especially considering the elderly patients who were possibly more affected by the OVCF than young patients. The cost of treating OVCF should also be considered in future studies. In 2013, Svedbom et al^[62]^ performed a cost-effectiveness analysis to compare balloon kyphoplasty, vertebroplasty, and nonsurgical management for the treatment of acute OVCF. The results revealed that PKP may be a cost-effective strategy for the treatment of OVCF. When considering the cost, some extra expenses should be included, such as nursing costs during the period of treatment.

### 4.2. Internal relations between the articles indicated the focus of the field and forecast the hotspot

The cluster categories of keyword co-citation indicated that the complications of vertebral augmentation were important to researchers. Although this kind of operation is minimally invasive, it could be associated with some severe complications such as fatal pulmonary embolism,^[67,68]^ cement leakage into the spinal canal, or catastrophic consequences caused by thermal necrosis (#8).^[63–66]^ Adjacent fractures^[69,70]^ were the most frequently discussed complications. The operation technique was an important issue in this field, since balloon kyphoplasty (#1) had been verified to be superior to PVP. Certainly, the indication of the operation was vital. This kind of operation might be suitable for older women (#7) when they were suffering from a vertebral compression fracture (#2). All the standards of this technology (#5) should be discussed seriously. Meanwhile, basic knowledge includes biomechanics (#3) and the material used to fill the vertebral body (#4), which could be considered to improve the result of the operations. All these trends can also be seen in the keyword with the strongest citation bursts. The keywords with the strongest citation bursts were augmentation and balloon kyphoplasty, which could be seen as an indication that researchers still had confidence in this technology, but there were still several workers before.

## 5. Conclusions

Despite the number of publications decreasing since 2009 after the publication of 2 authoritative level I evidence studies, there are still contrary opinions about PVP and PKP. These procedures are being implemented worldwide, especially for treating OVCFs in elderly patients. It appears that there will be a long way to go before the different opinions reach a consensus. PVP and PKP should be performed cautiously, including choosing patients strictly and informing patients and their families of the results and potential risks thoroughly. Meanwhile, new methods should be explored for further research, which might be more deliberate and objective. With these methods, more patients with different baselines should be selected for longer follow-up. Additionally, the evaluation factors should be more extensive, covering relative fields including the treatment results in short and long term, the long-term survival rate, the costs associated with the treatment of the patients, and the nursing costs borne by medical institutions or families.

## Acknowledgments

The authors of the current article acknowledge all authors listed meet the authorship criteria according to the latest guidelines of the International Committee of Medical Journal Editors and that all authors are in agreement with the manuscript.

## Author contributions

Conceptualization: Yu Chen, Shuang Wang, Liang Zhang, Jun-Xiong Ma PanData: Yu Chen, Shuang Wang Formal analysis: Shuang Wang, Liang Zheng Methodology: Yu Chen, Jun-Xiong Ma PanfSoftware: Hong Wang, Bo-Hua Zhang Visualization: Shuang Wang, Shao-Tong Sun, Xin-Lei Guo Writing-original draft: Shuang Wang, Liang Zheng, Jun-Xiong Ma PanWrition-review&editing: Yu Chen, Liang-Bi Xiang.
